# The German Multi-Dimensional Perceived Autonomy Support Scale for Physical Education: Adaption and Validation in a Sample of Lower Track Secondary School Students

**DOI:** 10.3390/ijerph17197353

**Published:** 2020-10-08

**Authors:** Julia Zimmermann, Henri Tilga, Joachim Bachner, Yolanda Demetriou

**Affiliations:** 1 Professorship of Educational Science in Sport and Health, Department of Sport and Health Sciences, Technical University of Munich, Georg-Brauchle-Ring 62, 80992 Munich, Germany; joachim.bachner@tum.de (J.B.); yolanda.demetriou@tum.de (Y.D.); 2 Institute of Sport Sciences and Physiotherapy, Faculty of Medicine, University of Tartu, Ujula 4 str., 51008 Tartu, Estonia; henri.tilga@ut.ee

**Keywords:** autonomy support, teaching, self-determination theory, questionnaire, self-efficacy, intrinsic value, bi-factor model, cognitive autonomy support, organizational autonomy support, procedural autonomy support

## Abstract

Teachers’ autonomy support (AS) in physical education (PE) has positive effects on students’ affective and behavioral outcomes in PE. Even though the existence of three different dimensions of AS, namely cognitive, organizational and procedural AS has been suggested in educational settings, there is a lack of multidimensional instruments for the assessment of autonomy-supportive teaching in PE. The aim of this study was to validate the German Multi-Dimensional Perceived Autonomy Support Scale for Physical Education (MD-PASS-PE). The sample comprised 1030 students of grades 6 through 10. Internal consistency was used to test the reliability of the assumed subscales. Factorial validity and measurement invariance across gender and age were examined by confirmatory factor analyses. Structural equation modeling was used to evaluate criterion validity. The subscales exhibited acceptable to good internal consistency. The assumed three-factor structure was confirmed within a bi-factor model including a general factor and three specific group factors. Results strongly supported measurement invariance across gender while tentatively suggesting measurement invariance across age. Criterion validity was supported as the MD-PASS-PE explained 15% and 14% of the variance in the constructs of self-efficacy and intrinsic value, respectively. The German MD-PASS-PE provides PE teachers with deeper insights into their autonomy-supportive teaching behavior, helping them to support their students’ autonomy in a holistic way.

## 1. Introduction

Teachers’ interpersonal style is of crucial importance in forming students’ psychological experiences both in the classroom and in physical education (PE) settings [1,2]. The way students perceive their interaction with their teachers may affect students’ intrinsic motivation, autonomous self-regulation, and several educational outcomes in psychomotor, cognitive, and affective learning domains [1,3,4,5,6,7]. Autonomy-supportive teaching is one of the most important aspects by which the interaction of teachers and students can be described [1]. This study aimed to provide a reliable and valid multi-dimensional instrument, which enables a deeper understanding of autonomy support in German PE classes.

The self-determination theory (SDT) [4] explains human motivation, emotions and personality processes in social contexts and has thus been intensively applied in PE [1,7]. The Basic Psychological Needs Theory (BPNT) [4,8] is one of the mini-theories included in the SDT framework. It postulates that teachers can influence students’ quality of motivation, well-being, and behavioral persistence by supporting students’ basic psychological needs for autonomy (i.e., experiencing a sense of volition), competence (i.e., experiencing a sense of effectiveness), and relatedness (i.e., experiencing a sense of connection) through need-supportive teaching [9,10,11,12]. In addition, need-supportive teaching relates positively to higher PE enjoyment [13], better performance [14], and higher physical activity levels in leisure time [15]. 

In the field of PE, the benefits of interacting with autonomy-supportive teachers are well documented [16]. Additionally, the effects of autonomy-supportive teaching have been examined about three times more often than the ones of competence or relatedness support [7]. Various effects of teacher autonomy support have been demonstrated with regard to students’ motivation for health behavior change [17], intention to be physically active outside the school setting [18], physical self-esteem [19] or experience of positive emotions [20,21].

There are various ways to support students’ need for autonomy, and different authors describe autonomy-supportive teaching in diverse ways [22,23,24,25,26]. In the framework of the SDT, the need for autonomy is characterized as a human desire to be the author of one’s actions and to experience a sense of volition and psychological freedom when engaging in an activity [23,27]. Thus, autonomy includes the pursuit of self-realization and self-determination that requires support from the social environment and interpersonal relationships. Within this theory, the provision of choice is regarded as one strategy to support autonomy [4,27,28]. Accordingly, autonomy-supportive teaching comprises more than just giving students a choice of different options [23]. To promote perceived self-regulation or intrinsic motivation, choice needs to be designed in such a way that it increases internal locus and volition [23]. Hence, teachers who successfully support their students’ need for autonomy, identify, nurture, and develop students’ motivational resources, like their interests, preferences, and personal goals [22,29]. Furthermore, autonomy-supportive teachers are able to convey an interpersonal message of support, understanding and the adoption of the students’ perspective, which students generally perceive as need-supportive [30].

Stefanou et al. [24] also argued that autonomy need not be synonymous with choice in a general way and proposed that there are multiple, distinct ways for teachers to support autonomy. Based on this assumption, Stefanou et al. [24] identified three manifestations of autonomy support in a learning environment, including cognitive choices as well as organizational and procedural choices. The first manifestation is called cognitive autonomy support (cAS), which promotes students’ responsibility for the learning process. This includes teaching behaviors like asking students to justify or argue their point, to generate their own solution paths or to evaluate their own and others’ solutions or ideas. cAS can further imply aspects such as the discussion of multiple approaches, strategies and ideas or the provision of informal feedback. Studies of autonomy-supportive teaching found that teachers offering higher rates of cAS tend to listen more, collect regular feedback from students, allow students to criticize the learning tasks, promote relevance, encourage students to think for themselves and answer students’ questions more often [22,31,32]. The second type of autonomy support is called organizational autonomy support (oAS), which can be considered as students’ responsibility to manage their learning environment. This includes aspects like developing rules together, choice of group members or seating arrangements. Tilga, Hein, and Koka [33] expanded this feature with regard to PE and further named the opportunities to choose equipment and exercise place as aspects of oAS. Finally, Stefanou et al. [24] characterized procedural autonomy support (pAS) as promoting students’ ownership about how to conduct the teaching and learning process. Teacher behaviors that are associated with pAS are, for example, providing the students with a choice regarding how competencies are demonstrated or with the possibility to present work in an individual manner. Tilga et al. [33] also added teaching behaviors like the explanation of a lesson structure and providing rationales.

Stefanou et al. [24] stated that cAS may be the essential component without which motivation and engagement may not be increased. Therefore, one might suppose that cAS is the most influential aspect of optimal learning. However, they further pointed out that oAS and pAS alone may not be sufficient, but may still be necessary to foster student engagement and intrinsic motivation. If students benefit from choice in all three aspects of autonomy support within regular lessons [24,34], a learning environment with high cAS, oAS and pAS may also be ideal for maximizing students’ engagement and intrinsic motivation in PE settings. 

Therefore, multidimensional measurement of the perceived autonomy-supportive teacher behavior is able to provide more detailed insights into effective teaching behavior in PE. By means of the Learning Climate Questionnaire (LCQ) [35,36], the Sport Climate Questionnaire (SCQ) [37] and the Perceived Autonomy Support Scale for Exercise Settings (PASSES) [38], autonomy support has been measured in a unidimensional way, equaling cAS [16,21,33]. Based on the lack of multidimensional scales to assess the perceived autonomy-supportive teacher behavior in PE settings, Tilga et al. [33] developed the Multi-Dimensional Perceived Autonomy Support Scale for Physical Education (MD-PASS-PE). Following the conclusions of Stefanou et al. [24], the items of the MD-PASS-PE were assigned to three categories (i.e., cognitive, organizational, and procedural autonomy support) with five items each to describe the autonomy-supportive teacher behavior in more detail. Confirmatory factor analysis supported the hypothesized three-factor solution, which was invariant across gender and age. The composite reliability coefficients of the cAS, oAS and pAS support subscales indicated good reliability (ρ between 0.83 and 0.89). Incremental criterion validity of the MD-PASS-PE compared to the unidimensional LCQ was examined with the satisfaction of the three BPN used as criterion variables. Both instruments significantly predicted need satisfaction. However, the MD-PASS-PE explained significantly more variance in competence need satisfaction than the LCQ [33].

To the best of the authors’ knowledge, there is no survey instrument in Germany that enables the assessment of students’ perceived autonomy support in PE in a multidimensional way. Therefore, the purpose of this study is to analyze the psychometric properties of the MD-PASS-PE with a sample of secondary school students from the least academically demanding track in Germany called *Mittelschule*.

## 2. Materials and Methods 

### 2.1. Participants

The sample of this study included 1030 students aged between 11 and 18 years (M = 13.4, SD = 1.48). The students attended grades 6 through 10 of the German *Mittelschule*. The *Mittelschule* represents the lowest educational level among secondary schools in Germany. The data was drawn from 67 classes of 10 schools (3 urban schools, 3 semi-rural schools, and 4 rural schools) and it involved 408 girls (39.6%) and 622 boys (60.4%). Of the participants, 51.8% indicated that German was the language spoken at home. Of the participants, 26.7% stated that another language was the one mainly spoken with their parents at home. The remaining 20.9% of the participants spoke German and another language to a comparable extent at home. Mean socioeconomic status (SES), calculated by the International Socio-Economic Index of Occupational Status (ISEI) [39] was 41.3 (SD = 12.8; *n* = 991) and thus clearly lower than in larger studies in Germany like the Programme for International Student Assessment (PISA) study in the year 2018, whose participants in grades 7 through 10 exhibited a mean SES of 51.8 (SD = 21.0; *n* = 4346). Body mass index (BMI = kg/m²) was on average 22.2 (SD = 4.8) which is within the healthy range of 18.5 to 24.9. The students participated in mandatory single-sex PE lessons for two hours a week.

### 2.2. Measures

#### 2.2.1. Pilot Study

The psychometric scales used in the main study were rigorously pilot tested in advance. The sample of the pilot study comprised 193 students of 11 classes from one urban and one rural German *Mittelschule* (grades 6 through 10). The main goal of the pilot study was to examine the applicability of the translated and adapted items as well as the scale format. Piloting was also used to test the general feasibility of a questionnaire study in this specific sample of academic underachievers mainly stemming from a low socioeconomic background.

While the students were completing the questionnaire, members of the assessment team marked the items that caused the students problems in understanding. After the questionnaire was completed, structured cognitive interviews [40] were conducted with two academically over-performing and two under-performing students compared with the level of each class. The interviews took place in a separate area, so that each student was able to express his/her opinion freely and independently from the classmates. The insights of the pilot study led to the final version of the questionnaire, in which the wording of the items was slightly adapted. Furthermore, based on the pilot study, a manual was prepared that should help the assessment team of the main study to answer consistently to the questions of the students regarding the respective items. 

#### 2.2.2. Autonomy Support by PE Teacher

• MD-PASS-PE Questionnaire Assessment

The autonomy-supportive behavior of the PE teachers was measured with the Multi-Dimensional Perceived Autonomy Support Scale for Physical Education (MD-PASS-PE) [33]. The MD-PASS-PE includes three subscales measuring cognitive, procedural and organizational autonomy support. Each subscale contains five items. An example item for cognitive autonomy support taken from the English version would be: “My PE teacher allows me to express my opinion”. “My PE teacher guides students in finding solutions” and “My PE teacher allows me to do exercises using different methods” are representative for the procedural and organizational autonomy support items, respectively. Students responded by means of a 7-point Likert scale from 1 = strongly disagree to 7 = strongly agree. The back-translation technique [41] was used to translate the items into German. First, the original items were translated from English into German by a team of researchers comprising bilingual native speakers and experts from the field of sports pedagogy. The translated German version was then back-translated into English by another team of bilingual native speakers. After comparing this version with the original scale, a remaining difference in one item between the versions was discussed and solved within a committee of bilingual researchers. The German version of the MD-PASS-PE is provided as supplementary material.

Former confirmatory factor analyses supported both a three-factor solution and a second-order model, which underlined the assumption of three conceptually distinct but highly related factors [33]. Further studies supported the reliability and factorial validity of the scale [42,43,44,45]. 

#### 2.2.3. Academic Self-Efficacy in PE

Academic self-efficacy in PE was assessed using a German 5-item scale originally designed to measure academic self-efficacy [46]. The items were adapted to the context of PE. Students responded on a 4-point Likert scale.

#### 2.2.4. Intrinsic Value of PE

To measure the intrinsic value that students ascribe to PE, a German 6-item scale including a 5-point Likert scale was used. The original scale was designed to measure the intrinsic value of mathematics and exhibited acceptable factorial validity and good internal consistency [47]. For the present study, the items were adapted to the PE context.

#### 2.2.5. Socioeconomic Status

To estimate their socioeconomic background, the students had to name and describe their parents’ jobs. The students’ answers were classified with regard to the International Socioeconomic Index of occupational status (ISEI), which is based on the International Standard Classification of Occupation 2008 (ISCO-08) [39]. If the jobs of both parents could be classified, the job with the higher ISEI value (HISEI) was used. The ISEI has a possible range from 10 to 89, with higher values indicating a higher socioeconomic status. Socioeconomic status could not be estimated for every student, because some students did not know or could not sufficiently describe their parents’ jobs. 

### 2.3. Procedures 

The study was conducted in accordance with the Declaration of Helsinki and was approved by the ethics commission of the Technical University of Munich (304/19 S) and the supervisory school authorities in charge. After approval of the ethics committee and the supervisory school authorities, the principals of the schools in the participating school district were contacted via phone and e-mail. They were provided with the documents informing the teachers, parents and students about the study. Additionally, they were sent the consent forms several weeks before the scheduled beginning of the study. The students did not participate unless they themselves as well as their parents, their PE teacher and the school principal had approved the participation. The students were not rewarded for participation in any form. They could leave out questions if they did not want to answer. They also had the possibility to withdraw their participation at any time before, during or after data collection without any personal consequences. 

The paper-and-pencil data collection took part within regular school lessons and lasted on average 35 min. To make sure that the PE constructs represented trait measures instead of state measures, data assessments did not take place directly after PE lessons. Before the beginning of the data collection, the head of the assessment team and one research assistant informed the students about the purpose and the procedure of the assessments. After the students were explained in detail how to handle the different response scales, they started to fill in the questionnaire on their own. The students could give a signal at any time and ask questions quietly in case of uncertainties. After a student had completed the questionnaire, he/she went outside of the classroom where height and weight were measured by means of a stadiometer and a digital weight scale by two other research assistants. These procedures applied to the preparation and implementation of both the pilot and main study. 

### 2.4. Statistical Analysis

SPSS Statistics and SPSS AMOS (Version 23.0; IBM Corp., Armonk, NY, USA) were used for the data analysis. Data screening showed that the study variables had less than 5% of missing values. The non-significant Little’s [48] test indicated that the data were missing completely at random (χ2 = 990.631, df = 950, *p* = 0.175). Since missing values inhibit structural equation modelling based on maximum likelihood estimation with 5000 bootstrap samples, which would be used later in the statistical analysis in AMOS, missing values had to be replaced [49,50,51]. Therefore, the Expectation Maximization algorithm was used to impute missings. The data was screened for univariate normal distribution based on the Byrne [49] recommendations, which consider values for skewness and kurtosis of each item ranging between −7 to +7 as acceptable. Internal consistency was estimated by means of Cronbach’s alpha coefficients. In a preliminary evaluation of the factor structure, an exploratory factor analysis (EFA) based on a principal component analysis was conducted. A direct oblimin rotation was used to account for the assumption of significantly related factors.

Fit of the proposed factor structure of the scales with the data was examined by means of confirmatory factor analysis (CFA). The following goodness-of-fit indices suggested by Hu and Bentler [52] were used to evaluate model fit: the comparative fit index (CFI), the Bentler–Bonett normed fit index (NFI), the Bentler–Bonett non-normed fit index (NNFI), and the root mean square error of approximation (RMSEA). An acceptable fit of the data with the hypothesized model is indicated by values ≥ 0.90 for the CFI, NFI and NNFI indices, and values ≤ 0.08 for the RMSEA index. Additionally, the items should exhibit standardized regression weights higher than 0.40 [53].

Measurement invariance across gender and age was tested by means of multi-group confirmatory factor analysis (MG-CFA). Thereby, the fit of one model is compared to the fit of another model in which one constraint is added. Thus, after an unconstrained model is introduced, more and more constraints are added in a stepwise manner, which assume factor loadings, item intercepts, latent variances and factor covariances to be equal across groups [49,54,55]. The commonly used comparative fit index (CFI) and the root mean square error of approximation (RMSEA) were considered for model comparison [56]. According to Chen [57], invariance is supported if the changes in CFI and RMSEA values are below 0.01 and 0.015, respectively, every time a new constraint is added. If the MG-CFA supports the assumption that factor loadings are equal across groups, weak measurement invariance is given. Strong measurement invariance requires both factor loadings and item intercepts to be equal across groups [58].

Self-efficacy and intrinsic value were used to examine criterion validity of the MD-PASS-PE. For this, structural equation modelling based on maximum likelihood estimation with 5000 bootstrap samples was used [49,50]. Based on the control-value theory (CVT) of learning and achievement emotions [59,60], the impact of the social environment (e.g., autonomy support) on students’ learning and achievement emotions should be mediated by the cognitive appraisals of subjective control and subjective values [59]. Subjective control relates to expectations and attributions about the perceived controllability of achievement-related actions and outcomes (e.g., self-efficacy expectations, which refer to one’s perception of his/her capacity to perform a learning task). Subjective values refer to the individual significance of achievement-related activities and outcomes (e.g., intrinsic value, which refers to the students’ beliefs about the learning activity itself). The effects of perceived teachers’ autonomy support on students’ self-efficacy and intrinsic value have been empirically assessed in different educational settings [61,62,63,64,65]. The findings indicated that autonomy-supportive teaching increases self-efficacy and intrinsic value. Hence, autonomy support should positively influence the two cognitive appraisals of self-efficacy and intrinsic value in PE settings as well.

## 3. Results

### 3.1. Preliminary Analysis

Univariate normal distribution of the MD-PASS-PE items was supported as skewness ranged between—1.94 and 0.68 and kurtosis between—1.07 and 3.25. Cronbach’s alpha values and the non-latent correlations between the scales are presented in Table 1. In the EFA, three factors with an eigenvalue > 1 were extracted, including a strong first factor. The extracted factors explained 54.62 % of the variance in the 15 items. Two items of the pAS and oAS exhibited cross-loadings.

### 3.2. Factorial Validity

**Figure 1:**
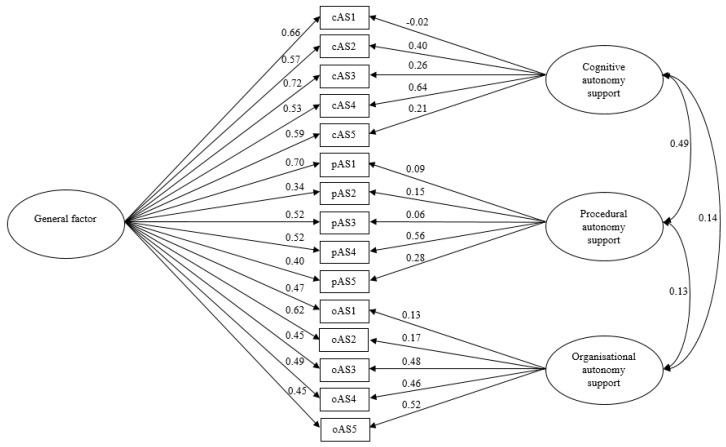
Bi-factor model of the German Multi-Dimensional Perceived Autonomy Support Scale for Physical Education (MD-PASS-PE). Standardized regression weights are presented next to the single-headed arrows, factor correlations are presented on the right of the double-headed arrows. Based on high modification indices, covariances were set between the error terms of items pAS3 and pAS5, and between the error terms of items oAS1 and oAS2.

With the first CFA, the assumed three-factor structure of the MD-PASS-PE was tested. Fit indices suggested an acceptable model fit (see Table 2, Model 1). Standardized regression weights ranged between 0.37 and 0.78 with one factor loading below 0.40. The correlations between the latent factors were strong and significant (0.77 ≤ r ≤ 0.94). Because the high factor correlations might suggest a unidimensional solution, the fit of a one-factor model was also tested. Goodness-of-fit indices did not indicate a satisfying model fit (see Table 2, Model 2). With regard to these findings, a bi-factor model with both a general factor, defined by all 15 items, and three specific group factors (cAS, pAS, and oAS), defined by the items of the respective subscales, was tested. Goodness-of-fit indices indicated a good model fit to the data (see Table 2, Model 3 and Figure 1). The standardized regression weights on the general factor ranged between 0.34 and 0.72 with one factor loading below 0.40. The factor loadings on the specific domains of autonomy support were mostly lower than in model 1 (−0.02 ≤ λ ≤ 0.64).

### 3.3. Measurement Invariance

To test for measurement invariance across gender, multigroup analyses examined the fit of the bi-factor model in girls’ and boys’ subsamples. When adding the constraints of equal factor loadings and item intercepts across gender groups, the changes in CFI and RMSEA were below 0.01 and 0.015, respectively. Thus, strong measurement invariance across gender was supported.

Measurement invariance across age groups was examined by comparing students of grades 6 and 7 (*n* = 490), representing the German “Unterstufe”, with students of grade 8 through 10 (*n* = 540), who belong to the “Mittelstufe”. Factor loadings and item intercepts of the bi-factor model were equal across groups, which speaks for strong measurement invariance across age groups. To compare groups with the largest age difference, another multigroup analysis was conducted including only sixth (*n* = 237, mean age = 11.73) and ninth graders (*n* = 230, mean age = 14.85). Students from grade 10 could not be selected for this comparison, as there were only 50 tenth graders taking part in the study. Changes in CFI were at 0.02 when introducing the constraint of equal factor loadings. Changes in RMSEA, however, were marginal and supported strong measurement invariance.

### 3.4. Criterion Validity

**Figure 2:**
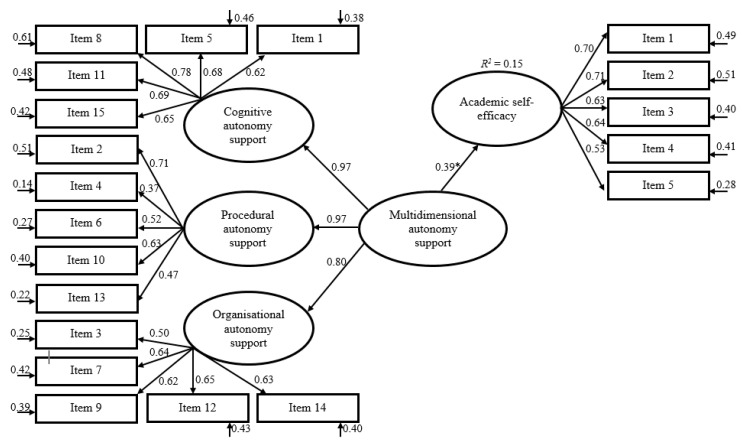
Structural equation modelling to predict academic self-efficacy in physical education (PE) from the latent multidimensional autonomy support of PE teachers. Including standardized regression weights. Regression weight of academic self-efficacy on multidimensional autonomy support is statistically significant (*p* < 0.001). R^2^ = coefficient of determination.

**Figure 3:**
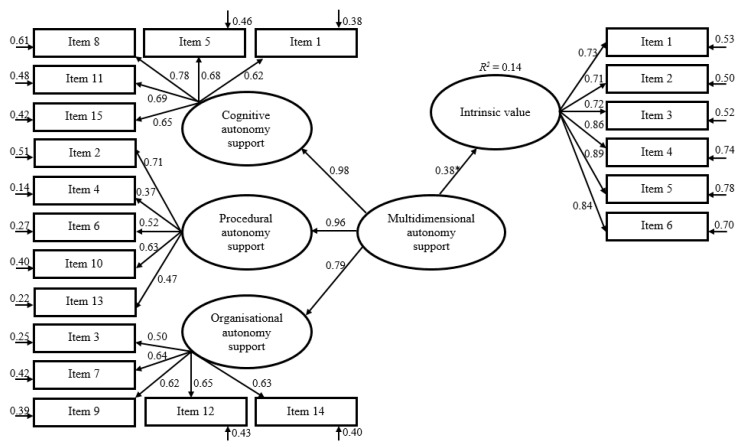
Structural equation modelling to predict intrinsic value of PE from the latent multidimensional autonomy support of PE teachers. Including standardized regression weights. Regression weight of intrinsic value on multidimensional autonomy support is statistically significant (*p* < 0.001). R^2^ = coefficient of determination.

To evaluate criterion validity, self-efficacy and intrinsic value were used as criterion variables in structural equation models. The latent criterion constructs were regressed on a latent multidimensional autonomy support factor, which was further regressed on the three specific autonomy support domains. In both cases, the multidimensional autonomy support factor served as a statistically significant predictor and was able to explain 15% and 14% of the variance in self-efficacy and intrinsic value, respectively (see Figure 2 and Figure 3).

## 4. Discussion

### 4.1. General Discussion

In this study, the MD-PASS-PE was adapted to the context of German PE lessons and was validated with regard to its internal consistency, structural validity and measurement invariance as well as its criterion validity. In a sample of German lower-track secondary school students, the three-factor structure could be replicated within a bi-factor model, thus supporting the existence of the three conceptually distinguishable but highly correlated factors cognitive, organizational and procedural autonomy support [33]. The German MD-PASS-PE offered acceptable to good levels regarding internal consistency. Results provided strong support towards measurement invariance across gender while tentatively suggesting measurement invariance across age. Criterion validity regarding constructs of subjective control and value was demonstrated.

After internal consistency analysis suggested that the subscales exhibited satisfying reliability and that each of them consistently measured one construct, the structural validity of the MD-PASS-PE was analyzed. CFA of the proposed three-factor structure showed an acceptable model fit (see Table 2, Model 1). However, together with the high correlations between the three latent factors, model fit indices gave reason to find solutions that fit the data better. To make sure that the three factors exhibited discriminant validity, the fit of the three-factor model was compared to the fit of a one-factor solution. The unidimensional model did not fit well to the data (see Table 2, Model 2), which indicated that by means of the MD-PASS-PE autonomy support is measured in a multidimensional manner. Finally, to reflect a factor structure that represents distinct constructs, which are still highly correlated, a bi-factor solution was modeled. In this model, variance in the manifest items should be explained both by a general latent factor and by a specific group factor representing the respective domain of autonomy support. The bi-factor model fitted well to the data (see Table 2, Model 3). The comparison of the fits of the tested models indicates that the constructs of cognitive, organizational and procedural autonomy support represent factors that are conceptually and empirically distinguishable but highly relate to each other. This interpretation was also supported in studies validating the MD-PASS-PE in samples of secondary school students in Estonia and Spain [33,42].

To account for high correlations between the three suggested aspects of autonomy support in the Estonian sample, Tilga et al. [33] tested a second-order model in which the three first-order factors (cAS, pAS, and oAS) were represented by one second-order factor. This model fitted the data equally well as a standard first-order three-factor model, which also supported the assumption of distinct but highly related autonomy support factors [33].

Although second-order and bi-factor models produce similar fits in various situations and suggest the same conclusions regarding factor structure, they implicate different features for further interpretations. In a second-order model, standardized path coefficients comprise both the direct influence from the first-order factor and mediated influences from the second-order factor [66]. The bi-factor model, however, is able to independently explain the direct effects of the general factor and the specific group factors on subtest performance, i.e., item scores. This way, it can be examined if an item rather represents a general trait or a more specific sub-trait [67]. Therefore, a bi-factor model is less ambiguous and thus more easy to interpret, as inferences regarding the influence of latent factors on item scores can be drawn immediately rather than using inferences from inferences, which are present in higher-order models [68,69,70]. In the present bi-factor model, standardized path coefficients of the general factor to the items were mostly higher than the ones of the specific group factors. This indicates a dominant general factor to which the unique effects of the specific group factors are added. The dominance of the general latent factor might be attributed to a possible deficit in the ability of lower track students to differentially rate their learning environment [71]. Instead, when completing the questionnaire, they might rather think of the choices they are provided with by their PE teacher during the lesson. Thus, the most likely interpretation of the general latent factor in the bi-factor model would be the overall autonomy support during PE in a sense of choice.

An alternative interpretation of the general latent factor would be an even more global evaluation of PE in terms of a rating from good to bad. Studies in the school context have shown that students in a given class come to highly similar ratings in scales assessing different aspects of lesson quality [47,72]. This suggests that students tend to evaluate lessons from a more emotional standpoint expressing their general approval of the lesson. In the case of the dominant general factor found here, one might therefore conclude that it represents a personal rating of lesson quality [47].

On the other hand, it has to be noted that the dominant general factor and the high correlations between the specific autonomy support factors do not necessarily have to be a consequence of a deficit in differentiation. With respect to the concepts of the specific autonomy support factors, high correlations should be expected. The teaching strategies used to support procedural autonomy [24] have a wide overlap with the teaching strategies for implementing structure [73], which is considered as an important part of the teaching process [74,75]. Furthermore, the unidimensional measurement of autonomy support widely represents the assessment of cAS [33]. In classroom and PE settings, a high correlation between autonomy support, usually measured in a unidimensional way, and the provision of the structure was reported [73,76]. Thus, it is not surprising that there are also high correlations between cAS and pAS.

Overall, the examination of the factorial validity of the MD-PASS-PE clearly speaks for a bi-factor model that includes a dominant general factor and three specific group factors. The group factors were highly correlated. This result was further supported by an exploratory factor analysis, which identified cAS as the most important group factor explaining most of the variance in the items. However, pAS and oAS substantially accounted for variance over and above the parts explained by cAS. Additionally, a unidimensional solution clearly exhibited the worst model fit. Summed up, variance in the manifest items is best explained by both a general factor, most likely representing overall autonomy support, and three distinct but highly related specific factors representing cAS, pAS and oAS.

The bi-factor model was used to check the invariance of the measurements across gender and age. Multigroup analyses supported the assumption of equality regarding factor loadings and item intercepts across gender. Hence, strong measurement invariance was fulfilled. It can thus be assumed that the measured constructs have the same conceptual meaning and that the association between the manifest items and the respective latent factor is the same regardless of the gender of the participant.

Results of the multigroup analyses concerning age clearly suggested strong measurement invariance when students of the German Unterstufe (grades 6–7) were compared to the ones of the Mittelstufe (grades 8–10). When sixth graders and ninth graders were compared, results were rather ambiguous. Changes in CFI values did not indicate equal factor loadings across groups, which might lead to the assumption that the cognitive development of students between grades 6 and 9 on this educational level impedes measurement invariance. Changes in RMSEA values, however, were small and clearly spoke for equal factor loadings and item intercepts, which indicated strong measurement invariance. This finding was unusual because CFI and RMSEA normally are equally sensitive to different factor loadings across groups [57]. Above all, with regard to the influence of the type of factor models or the types of invariance models being compared, the cut-offs for maximum change in model fit in invariance testing should not be considered as strict rules [77]. Therefore, multigroup analyses rather speak for measurement invariance across the age groups included in this study. However, these results need to be replicated and examined closer in future studies.

Structural equation models including academic self-efficacy in PE and intrinsic value of PE as criterion variables supported the criterion validity of the German MD-PASS-PE. About 15% of the variance in these variables was explained by a latent factor representing multidimensional autonomy support. This is in line with the assumptions of CVT [59] and empirical studies about the effect of autonomy support on self-efficacy and intrinsic value in educational settings other than PE [65].

### 4.2. Strengths and Limitations

This study provides a thorough validation of the German MD-PASS-PE in a large sample. A reliable and valid instrument for the multidimensional measurement of teacher autonomy support in PE is provided. Results of former studies with Spanish and Estonian samples were replicated to a large extent. By means of a bi-factor model, independent contributions of the general latent factor and the specific autonomy support factors to variance in item scores could be differentiated. Specific implications regarding the use of the MD-PASS-PE and the interpretation of the scores in a sample of academic underachievers are discussed.

Future studies should replicate the analyses concerning measurement invariance across age as results were ambiguous to some extent and because the range of the participants included in this study lay between grades 6 and 10. Additionally, measurement invariance across time could be examined in a longitudinal design. Furthermore, results should be replicated with students from schools with different levels of formal education. Lastly, a comparison of the MD-PASS-PE with an instrument measuring autonomy support in a unidimensional way in terms of their predictive abilities of school-related psychological constructs would be interesting.

## 5. Conclusions

Numerous effects of teacher autonomy support have been demonstrated with regard to positive affective and behavioral outcomes in PE [17,18,19,21]. This validation of the German MD-PASS-PE not only provides a reliable and valid measurement tool for autonomy support in PE, but also supports the assumption that teachers’ autonomy support can be provided in a multifaceted way [22,23,24,26]. Although different authors suggested different strategies of how to support autonomy in classroom and PE settings, the construct of autonomy was usually measured in a unidimensional way (e.g., LCQ) [33,42]. In this study, it was shown that cognitive, procedural and organizational aspects of autonomy support can be differentiated in PE lessons. This offers PE teachers the possibility to gain deeper and more exact insights into their teaching behavior. Furthermore, as a feedback tool, this instrument may help them in supporting their students in a holistic way as optimal learning benefits can be expected if cAS as well as pAS and oAS are provided [24].

## Figures and Tables

**Table 1:** Cronbach’s alpha coefficients and correlations between scales.

	cAS	oAS	pAS	SE	iVa
cAS	0.81				
oAS	0.61 *	0.76			
pAS	0.66 *	0.54 *	0.72		
SE	0.32 *	0.25 *	0.23 *	0.78	
iVa	0.36 *	0.20 *	0.23 *	0.61 *	0.91

**Note.** cAS = cognitive autonomy support; oAS = organizational autonomy support; pAS = procedural autonomy support; SE = academic self-efficacy in PE; iVa = intrinsic value of PE; * = correlation is statistically significant (*p* < 0.001); Cronbach’s alpha values of the scales are presented in italics.

**Table 2:** Fit indices for confirmatory factor models.

Model			
Model Parameter	Model 1: Three-Factor Model	Model 2: One-Factor Model	Model 3: Bi-Factor Model
χ2	499.04	678.66	281.97
df	85	88	71
CFI	0.921	0.887	0.960
NFI	0.906	0.872	0.947
NNFI	0.902	0.865	0.940
RMSEA	0.069	0.081	0.054

**Note.** χ2 = chi-squared; df = degrees of freedom; CFI = comparative fit index; NFI = Bentler–Bonett normed fit index; NNFI = Bentler–Bonett non-normed fit index; RMSEA = root mean square error of approximation.

## Supplementary Materials

The following are available online at https://www.mdpi.com/1660-4601/17/19/7353/s1, data set S1: data set.
